# Fixation method can affect posterior tibial slope in opening-wedge high tibial osteotomy: a retrospective study

**DOI:** 10.1186/s13018-023-04281-8

**Published:** 2023-10-17

**Authors:** Hamid Reza Yazdi, Ali Torkaman, Amir Ebrahimzadeh Babaki, Mohammad Soleimani, Arvin Eslami

**Affiliations:** 1 Department of Orthopedic, School of Medicine, Iran University of Medical Sciences, Tehran, Iran; 2 Bone and Joint Reconstruction Research Center, Department of Orthopedics, School of Medicine, Iran University of Medical Sciences, Tehran, Iran; 3 Department of Epidemiology, School of Medicine, Iran University of Medical Sciences, Tehran, Iran

**Keywords:** High tibial osteotomy, TomoFix, Puddu, Posterior tibial slope

## Abstract

**Background:**

Posterior tibial slope (PTS) alterations following open-wedge high tibial osteotomy (OWHTO) can cause instability and excessive tibial translation in the sagittal plane. These changes can be influenced by the type of fixation. This study aims to compare PTS changes between patients undergoing OWHTO with Puddu plate or TomoFix plate fixation.

**Methods:**

In this retrospective cohort study, we included 104 knees from 85 patients undergoing OWHTO, with a mean age of 41.98 ± 9.95 years; 51.8% of the participants were male. Seventy-two knees were fixed with Puddu plates, while 32 knees were fixed with TomoFix plates. PTS changes, demographic factors, Cincinnati Knee Rating Score (CKRS), Tegner-Lysholm score (TLS), length of stay (LOS), and complications were evaluated. PTS changes were measured preoperatively, immediately postoperatively, and at the 6-month follow-up.

**Results:**

Demographic factors were similar between the Puddu plate and TomoFix groups. There were no significant differences in preoperative, postoperative, or follow-up PTS measurements between the two groups. PTS changes were not significant in the TomoFix group postoperatively or at follow-up. However, the Puddu plate group showed a significant increase in PTS both postoperatively (*P* = 0.027) and at follow-up (*P* = 0.014). CKRS, TLS, LOS, and complications did not significantly differ between the groups.

**Conclusion:**

While overall PTS changes did not significantly differ between the Puddu Plate and TomoFix Plate groups, analyzing changes within each group revealed distinct results. TomoFix fixation exhibited nonsignificant PTS changes, while Puddu plate fixation resulted in a significant increase in PTS after surgery and at the 6-month follow-up. Our findings suggest that the choice of fixation may influence PTS changes after OWHTO.

**Level of Evidence**: Level III.

## Introduction

Medial knee osteoarthritis (OA) is a prevalent joint disorder worldwide, presenting a therapeutic challenge, especially in young, active patients [1]. High tibial osteotomy (HTO) has emerged as a well-established procedure for addressing genu varus and medial tibiofemoral OA in this patient population [1, 2]. Additionally, HTO is a feasible treatment option in mild-to-moderate medial compartmental OA, particularly among elderly individuals with high functional demands [3].

Alterations in posterior tibial slope (PTS) and patellar height have been associated with HTO [4, 5]. Specifically, open-wedge high tibial osteotomy (OWHTO) has been shown to increase PTS [6, 7]. Such changes in PTS can influence knee biomechanics, potentially leading to instability and excessive tibial translation in the sagittal plane, which may contribute to the progression of OA [8]. An increase in tibial slope can result in restricted extension and, in some patients, exert undue pressure on the anterior cruciate ligament [9]. Several factors can contribute to this slope increase, including technical considerations and the potential influence of the chosen load fixation device. Furthermore, an increase in PTS may result in correction loss [10].

HTO leads to a highly unstable structure of the proximal tibia, which is the potential source of mechanical failure of plates and screws. Consequently, the use of fixation devices and optimal designs are essential to the success of HTO, especially for overweight or full-weight-bearing patients. Currently, several commercial implants are available for treating medial knee joint OA, including TomoFix small stature, TomoFix standard, Contour Lock, iBalance, and second-generation PEEK Power [11, 12]. Locking screws play a vital role in stabilizing the construct and reducing stresses on the implant and bone [13]. The choice of fixation method and the use of locking screws can significantly impact PTS changes [14]. When employing the Puddu plate, the inclusion of a metal block introduces the possibility of greater anterior cortex opening, potentially heightening the risk of an increased slope. Furthermore, fixation with the Puddu plate may offer less rigidity compared to TomoFix, potentially increasing the likelihood of posterior or anterior cortex collapse. Consequently, our aim is to investigate whether the choice of fixation has a discernible effect on early or late changes in tibial slope. For clarification, “early increase” denotes changes occurring concurrently with the surgical procedure, while “late increase” refers to alterations observed during the follow-up period. The relative lack of rigidity associated with Puddu plate fixation introduces the possibility of slope changes manifesting during the follow-up phase. Various studies have consistently reported superior outcomes with the use of TomoFix compared to other plates, particularly the Puddu plate [7, 14]. However, conflicting reports indicate no significant difference in slope changes between the Puddu plate and TomoFix [13].

As there is currently no literature addressing this specific inquiry, we embarked on the design of this study to investigate the potential impact of fixation method choice on early and late changes in tibial slope. Our hypothesis posits that the type of fixation device can contribute to alterations in tibial slope both shortly after surgery and during the follow-up period.

The principal aim of this investigation was to compare alterations in PTS and functional outcomes among patients who underwent OWHTO for medial tibiofemoral OA of the knee. We assessed the effectiveness of two distinct fixation systems: the nonlocking Puddu plating system (Arthrex, Naples, Florida) and the TomoFix plating system (DePuy Synthes, Oberdorf, Switzerland). Furthermore, we examined the influence of these fixation methods on PTS changes and the incidence of surgical complications.

## Material and methods

This retrospective cohort study included patients with medial compartment OA of the knee who underwent OWHTO at our University Hospital in Tehran, Iran, from January 2012 to March 2022. Ethical approval was obtained from the Ethics Committee of Iran University of Medical Sciences (approval number: 24014), and written informed consent was obtained from all patients. We utilized G*Power version 3.1 software (Dusseldorf, Germany) to perform the sample size calculation, which indicated that a total sample size of 102 would be sufficient for this study. Out of 150 patients initially reviewed, 65 were excluded, and 85 patients met the inclusion criteria for the study. The inclusion criteria were age between 18 and 65 years, undergoing OWHTO with either a Puddu plate or TomoFix, and a minimum follow-up of 12 months. Patients with concomitant surgeries, less than 12 months of follow-up, or missing pre- or postoperative radiographs were excluded.

The two groups received equivalent postoperative care and follow-up. Radiographic grading of knee OA was assessed using the Kellgren–Lawrence (K–L) scale [4]. Indications for OWHTO included medial knee pain, varus mechanical axis, OA K–L grades 1–3 for the medial compartment, and K–L grades 0–1 for patellofemoral (PF) or lateral tibiofemoral joints on radiographs. Patients with significant arthritic changes in the lateral compartment and/or PF joint on radiographs were not enrolled for HTO.

Demographics of patients, including age, sex, length of hospitalization, and correction degree during surgery, were extracted from the hospital records.

### Surgical procedure

All surgeries were performed by two fellowship-trained senior knee surgeons in one center with the same surgical technique. Although the patient selection, preoperative planning, surgical approach, and technique were consistent between the two surgeons, there was a difference in the fixation method. Specifically, one surgeon performed osteotomies in a single plane for all patients, while the other surgeon performed osteotomies in two planes. The choice of plate used for fixation was based on each surgeon’s preference. TomoFix fixation involved the use of locking screws, while Puddu plate fixation utilized nonlocking screws. The Miniaci preoperative planning technique was used to determine the correction angle [15].

All patients underwent preparation and draping under spinal anesthesia, with the patients in a supine position and a tourniquet applied. After surgery, a long leg splint was utilized, and all patients in both groups were ambulated with the help of a walker for nonweight bearing on the day after surgery. This nonweight-bearing status was followed for a period of 6 weeks. Follow-up visits were scheduled at two weeks, six weeks, three months, and six months postoperatively, during which radiographs were obtained. Postoperative care and rehabilitation protocols were identical for both fixation groups.

### Outcome measures

**Fig. 1:**
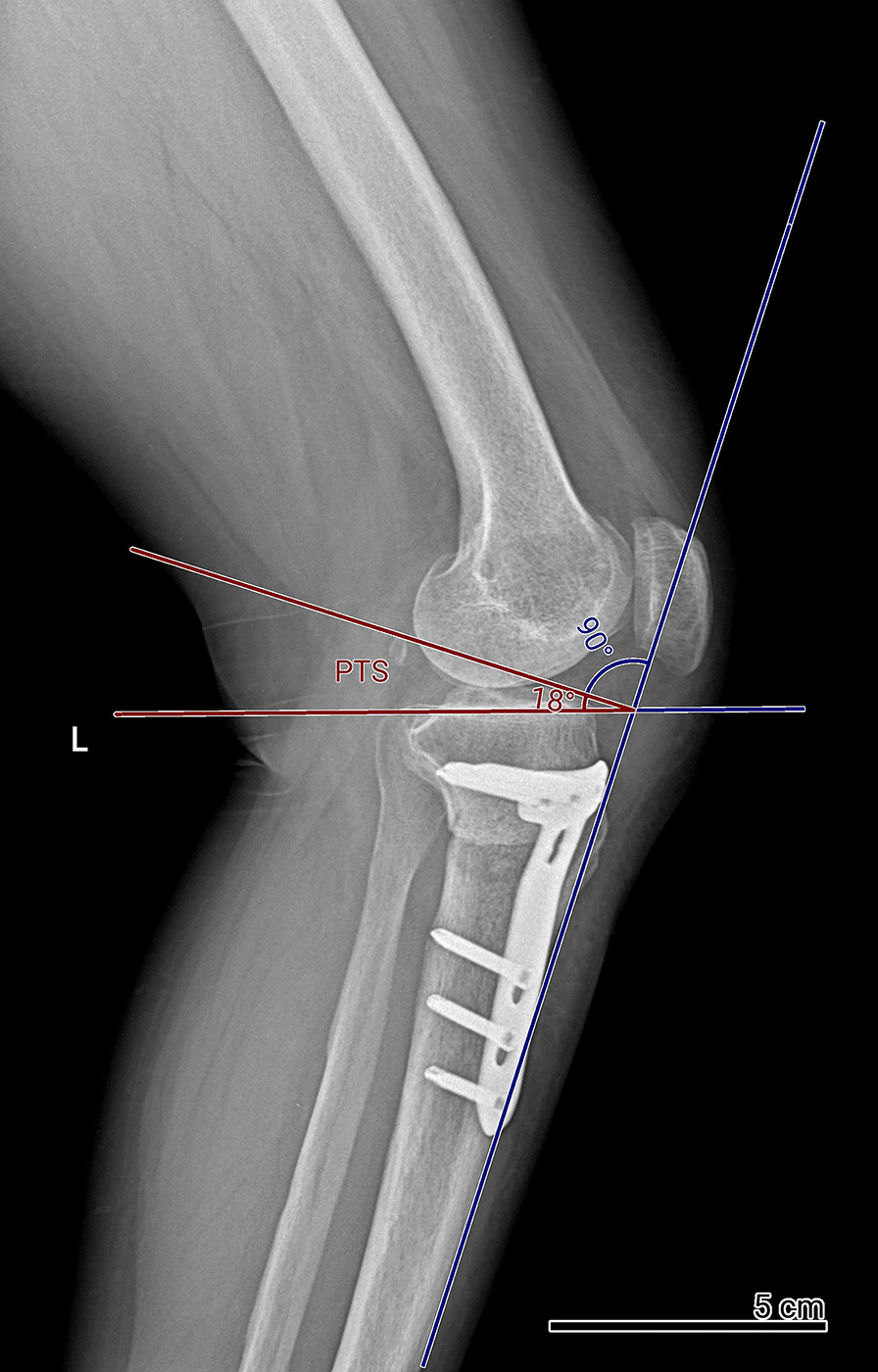
PTS measurement method in the radiograph

Radiographs were evaluated using the diVision system (MarcoPacs, Tehran, Iran). PTS was assessed in true lateral (showing congruent femoral condyles) long knee radiographs preoperatively, immediately postoperatively, and six months postoperatively. The measurements were conducted by two chief orthopedic residents under the supervision of a knee fellowship surgeon. The PTS measurement involved drawing a horizontal line perpendicular to the anterior tibial cortex, and a second line was drawn along the medial tibia plateau. The angle formed by the horizontal perpendicular line and the proximal tibial margin represents the PTS angle **(**Fig. 1**)** [16].

Lateral hinge fracture, nonunion, and malunion were also evaluated in the radiographs. Nonunion was defined as insufficient healing persisting for over 9 months, while delayed union was considered when insufficient healing persisted for more than 4 months [17]. The knee’s range of motion (ROM) was evaluated by two chief orthopedic residents using clinic records at a minimum of six months postoperatively. Additionally, information regarding height, weight, smoking status, underlying diseases (heart, kidney, thyroid, and diabetes), pain levels, the desire for device removal, the Modified Cincinnati Knee Rating System (CKRS) score, and the Tegner-Lysholm Knee Scoring Scale score was collected through phone call. Validated and translated questionnaires were utilized for data acquisition in this study [4, 5].

### Statistical analysis

Continuous variables are presented as the mean and standard deviation (SD), while categorical variables are expressed as the frequency and percentage. PSS version 26 software was used for data analysis. The relationship between two categorical variables was assessed using the chi-square test or Fisher’s exact test. For comparing continuous variables, the independent samples t test or Mann‒Whitney U test was utilized. Paired t tests or Wilcoxon signed-rank tests were employed to compare the changes within groups. A significance level of *P* < 0.05 was considered statistically significant.

The reliability of the measurements, assessed using the single-measure two-way random models for the interclass correlation coefficient (ICC), is presented in Table 1.

**Table 1:** Reliability assessment of tibial slope measurements using ICC

Angle	ICC	Lower bound	Upper bound
PTS pre-op	0.955	0.935	0.970
PTS post-op	0.955	0.934	0.969
PTS follow-up	0.956	0.936	0.970

## Result

The study included a total of 104 knees, with 72 knees fixed with Puddu plates and 32 knees fixed with TomoFix plates. The follow-up duration ranged from 12 to 112 months. Among the 85 patients, the mean age was 41.98 ± 9.95 years, and 44 (51.8%) were male. The mean BMI was 26.95 ± 3.91 kg/m^2^, and 18 patients underwent bilateral HTO (Table 2).

**Table 2:** Comparison of demographic factors, PMH, and follow-up between the two groups

Variable	Puddu plate	TomoFix	*P* value
Sex			0.649
Male	31 (53.4%)	13 (48.1%)	–
Female	27 (46.6%)	14 (51.9%)	
Age (year)	42.43 ± 10.02	41.03 ± 9.93	0.551
Side			0.492
Right	33 (45.8%)	17 (53.1%)	–
Left	39 (54.2%)	15 (46.9%)	
BMI	27.32 ± 4.18	26.17 ± 3.19	0.209
PMH	6 (10.3%)	2 (7.4%)	0.682
Smoking	10 (16.9%)	1 (3.7%)	0.088
Mean follow-up (month)	26.51 ± 8.38	28.84 ± 10.66	0.279

*BMI* Body mass index, *PMH* Past medical history

The comparison of PTS changes between the two groups revealed no significant difference before surgery (*P* = 0.554), after surgery (*P* = 0.596), or during the follow-up phase (*P* = 0.519). However, analyzing each group separately, the TomoFix group exhibited nonsignificant PTS changes postoperatively (*P* = 0.201) and at the final follow-up (*P* = 0.129), while the Puddu plate group showed significant PTS changes preoperatively (*P* = 0.027) and at the 6-month follow-up (*P* = 0.014) (Table 3).

**Table 3:** PTS changes in the two groups

PTS	Preoperative	Postoperative	*P* value*	Follow-up	*P* value^†^	*P* value^‡^
Puddu plate	16.27 ± 4.18	17.07 ± 4.22	0.027	17.12 ± 4.08	0.014	0.404
TomoFix	16.86 ± 4.69	17.71 ± 5.38	0.201	17.85 ± 5.23	0.129	0.259

*PTS* Posterior tibial slope

*Difference between preoperative and postoperative PTS

^†^Difference between preoperative and follow-up PTS

^‡^Difference between postoperative and follow-up PTS

In total, 20 knees with complications were observed. There was no significant difference in the complications between the Puddu plate and TomoFix groups, including lateral hinge fracture, nonunion, need for device removal, and tenderness (Table 4).

**Table 4:** Comparison of surgical complications between the two groups

Complication	Puddu plate (*n* = 72)	TomoFix (*n* = 32)	*P* value
Lateral hinge fracture	5 (6.9%)	4 (12.5%)	0.352
Nonunion	1 (1.4%)	1 (3.1%)	0.552
Need for device removal	2 (2.8%)	4 (12.5%)	0.050
Tenderness	4 (5.6%)	3 (9.4%)	0.473

The mean correction for all patients was 8.78 ± 1.32, with a mean length of hospital stay of 4.81 ± 1.55 days. Moreover, the mean CKRS and Tegner-Lysholm scores were 86.11 ± 12.67 and 88.35 ± 11.77, respectively. No significant differences were found in the comparison of correction, length of stay, CKRS, and Tegner-Lysholm scores between the two fixation groups (Table 5).

**Table 5:** Comparison of correction, length of stay, Cincinnati knee rating system score, and Tegner-Lysholm score between the two groups

Variable	Puddu plate	TomoFix	*P* value
Correction (degree)	8.93 ± 1.12	8.42 ± 1.65	0.092
Length of stay (days)	4.93 ± 1.64	4.56 ± 1.34	0.299
CKRS Score	87.62 ± 12.14	82.71 ± 13.35	0.089
Tegner-Lysholm Score	89.87 ± 11.40	84.93 ± 12.06	0.055

*CKRS* Cincinnati knee rating system

Regarding underlying diseases, eight patients had hypothyroidism, three had diabetes mellitus, one had colon cancer, one had breast cancer, one had multiple sclerosis, and one had hypertension. Only hypothyroidism was considered for analysis due to the small number of patients with other conditions. There was no statistically significant difference in PTS changes during surgery in people who had hypothyroidism compared to other patients (*P* = 0.983).

## Discussion

In this 10-year retrospective study, we compared the use of Puddu and TomoFix plates in patients undergoing OWHTO for medial knee OA. Our findings revealed no significant difference in PTS changes between the two fixation groups, and functional outcomes were similar in both groups. PTS alterations following OWHTO can impact knee biomechanics, leading to instability and excessive tibial translation in the sagittal plane, potentially contributing to OA progression. While OWHTO aims to correct coronal plane deformities, it can result in adverse sagittal plane events, including increased PTS, which may lead to correction loss [6, 7].

Several authors have highlighted that TomoFix provides superior outcomes compared to other plates, particularly the Puddu plate [7, 14]. A recent meta-analysis comparing survival, plate-related complications, and functional and radiological outcomes of Puddu or TomoFix plating systems showed that both systems could delay the need for arthroplasty. However, despite both implants yielding satisfactory functional outcomes, TomoFix demonstrated superior survival rates and a lower incidence of complications [18]. Golovakha et al. studied four groups, including the Puddu plate, TomoFix, bone graft, and a control group and found that TomoFix yielded the best outcome and correction, while the weakest results were associated with the Puddu plate [19]. Izaham et al. evaluated patients undergoing HTO with TomoFix and Puddu plates, demonstrating significantly higher stability with TomoFix [20]. Stoffel et al. compared patients undergoing HTO with TomoFix or Puddu plating systems and found that the TomoFix system provides higher stability than the Puddu plate [21]. In our study, the TomoFix group showed nonsignificant PTS changes, while the Puddu plate group exhibited significant PTS changes after surgery and at the 6-month follow-up. This can indicate the higher stability of TomoFix fixation.

Nevertheless, some prior studies reported that the plate type did not affect complication rates after HTO. Woodacre et al. found similar complication rates between patients using TomoFix and Puddu plates [22]. Another study by Kim et al., including patients undergoing HTO with TomoFix, Puddu plate, and Aescular plates, showed no significant difference in outcomes or complications among the three groups [23]. In our study, no significant difference in complications, such as lateral hinge fracture, nonunion, need for device removal, and tenderness, was observed between the TomoFix and Puddu plate groups.

The underlying reasons for PTS alterations following HTO have been subject to investigation. Several suggestions have been proposed, including the angulation of the slightly posterior center of rotation, attributed to the triangular shape of the proximal tibia with an anterior apex [24], and the challenge in sufficiently releasing the posterior soft tissue due to the presence of muscles and vessels [7]. Nerhus et al. demonstrated that fixing the plate near the posteromedial corner yields minimal or no PTS increase after OWHTO. Careful preoperative planning is essential to minimize or prevent PTS alterations in HTO procedures [25]. Our results align with the literature, suggesting that TomoFix fixation does not significantly alter PTS, while the Puddu plate increases PTS. Thus, TomoFix provides higher stability than the Puddu plate in OWHTO procedures.

## Strengths and limitations

Our study has several strengths, including the involvement of two fellowship-trained knee surgeons who performed all surgeries using the same technique and surgical approach. The demographic factors and patient selection were consistent between the two groups, and postoperative care, rehabilitation, and follow-up were standardized for both groups.

However, the study also has limitations. First, its retrospective design may introduce potential bias, and there is a relatively low amount of follow-up data available. Second, the surgeries were performed by two different surgeons, with one using a single-plane osteotomy technique and the other using a two-plane technique. Although both surgeries were conducted in the same center with consistent postoperative care, this difference in technique may have impacted the surgical outcomes. The use of locking screws in TomoFix fixation and nonlocking screws in Puddu plate fixation could also contribute to variations in PTS changes between the two groups. Additionally, the study did not include a comparison of bone density between the groups, which could potentially influence surgical outcomes. Despite these limitations, our findings provide valuable insights into the comparison of PTS changes and functional outcomes between patients undergoing high tibial osteotomy with Puddu or TomoFix plates, contributing to the existing knowledge in this field. Further studies, ideally randomized controlled trials, are warranted to address these limitations and could confirm our results.

## Conclusion

In our study, no significant difference was observed in PTS changes between patients undergoing HTO with Puddu plate fixation and those with TomoFix fixation. However, the Puddu plate group showed a significant increase in PTS in both immediate postoperative and 6-month follow-up radiographs, while the TomoFix group exhibited nonsignificant PTS changes. These findings suggest that TomoFix may provide superior stability compared to Puddu plate fixation after HTO. The incidence of complications and length of hospital stay were similar between the two groups. Further studies with a larger sample size are recommended to explore potential significant differences in greater detail.

## Data Availability

The datasets generated during the current study are available from the corresponding author upon reasonable request.

## Abbreviations

CKRS: Cincinnati Knee Rating Score
HTO: High tibial osteotomy
K–L: Kellgren–Lawrence
LOS: Length of stay
OA: Osteoarthritis
OWHTO: Open-wedge high tibial osteotomy
PF: Patellofemoral
PTS: Posterior tibial slope
ROM: Range of motion
SD: Standard deviation
TLS: Tegner-Lysholm score
